# GC-MS Based Identification of the Volatile Components of Six *Astragalus* Species from Uzbekistan and Their Biological Activity

**DOI:** 10.3390/plants10010124

**Published:** 2021-01-08

**Authors:** Haidy A. Gad, Nilufar Z. Mamadalieva, Stefan Böhmdorfer, Thomas Rosenau, Gokhan Zengin, Rano Z. Mamadalieva, Nawal M. Al Musayeib, Mohamed L. Ashour

**Affiliations:** 1 Department of Pharmacognosy, Faculty of Pharmacy, Ain Shams University, Cairo 11566, Egypt; haidygad@pharma.asu.edu.eg; 2 Department of Pharmacognosy, Faculty of Pharmacy, King Salman International University, South Sinai, Ras Sidr 46612, Egypt; 3 Institute of the Chemistry of Plant Substances, Academy of Sciences of RUz, Mirzo Ulugbek Str. 77, Tashkent 100170, Uzbekistan; nmamadalieva@yahoo.com; 4 Department of Bioorganic Chemistry, Leibniz Institute of Plant Biochemistry, Weinberg 3, D-06120 Halle (Saale), Germany; 5 Department of Chemistry, Institute of Chemistry of Renewable Resources, University of Natural Resources and Life Sciences, Vienna (BOKU University), Konrad-Lorenz-Straße 24, 3430 Tulln, Vienna, Austria; stefan.boehmdorfer@boku.ac.at (S.B.); thomas.rosenau@boku.ac.at (T.R.); 6 Biochemistry and Physiology Research Laboratory, Department of Biology, Science Faculty, Selcuk University, 42130 Konya, Turkey; gokhanzengin@selcuk.edu.tr; 7 Kokand State Pedagogical Institute, Turon Str. 23, Kokand 713000, Uzbekistan; rmamadalieva@yahoo.com; 8 Department of Pharmacognosy, College of Pharmacy, King Saud University, Riyadh 11495, Saudi Arabia

**Keywords:** leguminosae, *Astragalus*, chemometrics, GC-MS, volatile components, antioxidants, enzyme inhibition

## Abstract

The compositions of volatile components in the aerial parts of six *Astragalus* species, namely *A. campylotrichus* (*Aca*), *A. chiwensis* (*Ach*), *A. lehmannianus* (*Ale*), *A. macronyx* (*Ama*), *A. mucidus* (*Amu*) and *A. sieversianus* (*Asi*), were investigated using gas chromatograph-mass spectrometry (GC-MS) analysis. Ninety-seven metabolites were identified, accounting for 73.28, 87.03, 74.38, 87.93, 85.83, and 91.39% of *Aca*, *Ach*, *Ale*, *Ama*, *Amu* and *Asi* whole oils, respectively. Sylvestrene was the most predominant component in *Asi, Amu* and *Ama*, with highest concentration in *Asi* (64.64%). In addition, (*E*)-2-hexenal was present in a high percentage in both *Ale* and *Ach* (9.97 and 10.1%, respectively). GC-MS based metabolites were subjected to principal component analysis (PCA) and hierarchal cluster analysis (HCA) to explore the correlations between the six species. The PCA score plot displayed clear differentiation of all *Astragalus* species and a high correlation between the *Amu* and *Ama* species. The antioxidant activity was evaluated in vitro using various assays, phosphomolybdenum (PM), 2,2 diphenyl-1-picryl-hydrazyl-hydrate (DPPH), 2,2-azino bis (3-ethylbenzothiazoline-6-sulphonic acid) (ABTS), cupric reducing antioxidant capacity (CUPRAC), ferric reducing power (FRAP) and ferrous ion chelation (FIC) assays. In addition, the potential for the volatile samples to inhibit both acetyl/butyrylcholinesterases (AChE, BChE), α- amylase, α-glucosidase and tyrosinase was assessed. Most of the species showed considerable antioxidant potential in the performed assays. In the DPPH assay, *Ama* exhibited the maximum activity (24.12 ± 2.24 mg TE/g sample), and the volatiles from *Amu* exhibited the highest activity (91.54 mgTE/g oil) in the ABTS radical scavenging assay. The effect was more evident in both CUPRAC and FRAP assays, where both *Ale* and *Ama* showed the strongest activity in comparison with the other tested species (84.06, 80.28 mgTE/g oil for CUPRAC and 49.47, 49.02 mgTE/g oil for FRAP, respectively). *Asi* demonstrated the strongest AChE (4.55 mg GALAE/g oil) and BChE (3.61 mg GALAE/g oil) inhibitory effect. Furthermore, the best tyrosinase inhibitory potential was observed for *Ale* (138.42 mg KAE/g). Accordingly, *Astragalus* species can be utilized as promising natural sources for many medicinally important components that could be tested as drug candidates for treating illnesses such as Alzheimer’s disease, diabetes mellitus and oxidative stress-related diseases.

## 1. Introduction

*Astragalus* (family Leguminosae) is a large genus widely distributed in the temperate regions of the world, principally in Eurasia and North America. This genus is notable because of its worldwide taxonomic problems. Therefore, it represents an endless mine for taxonomists’ work that results in constant revision, particularly at the section level. *Astragalus* is a characteristic Irano-Turanian region genus, and many of its species show a narrow geographic range (narrow endemics), which makes them vulnerable to extinction. Most members of this genus are generally associated with semi-arid and arid habitats across the world [1]. There are about 650 species of *Astragalus* in the flora of Central Asia, while nearly 275 species are among the Uzbek flora [2].

Many *Astragalus* species have been used for centuries in the traditional medicine of Iran, Pakistan, India, China, and Korea. They have contributed in curing many illnesses, such as liver and kidney associated diseases, gastrointestinal disorders, and many others [1]. However, the most fame for many *Astragalus* species was gained owing to their potential to produce gum tragacanth, which has a number of well documented medicinal uses. In addition, it is an important substance in both the food and pharmaceutical industries [1].

Among these species, the roots of *Astragalus membranaceus* Bge. var. *mongholicus* (Bge.) Hsiao and *A. membranaceus* (Fisch.) Bge. along with their products are documented in the Chinese Pharmacopeia for the treatment of many syndromes associated with “qi deficiency”. On the other hand, in traditional Uzbek medicine, the decoction obtained from the fruits of *A. sieversianus* is used to remove kidney and bladder stones. Meanwhile, the ingested seeds are used to improve hernias in kids and are smoked to treat syphilis [2]. Leaves and gummy exudates of *A. rubrivenosus* Gontsch and *A. abolinii* Popov are considered useful in the treatment of kidney disease, hypertonic disease, and in burns as a demulcent [1].

Some members of *Astragalus* are well known for their pharmacological properties. It was reported that many species and their isolated components possessed promising anti-inflammatory, immunostimulant, antioxidant, anti-cancer, antidiabetic, hepatoprotective, cardioprotective, antibacterial, and antiviral activities [3]. Phytochemical and biological investigations of *Astragalus* have resulted in the isolation and identification of triterpenoid and steroidal saponins, flavonoids, phenylpropanoids, alkaloids, and some other compounds. About 260 secondary metabolites were isolated from the genus. They represent a very good source for drug discovery since most of the isolated saponins are found only in this genus, offering excellent lead drugs [4]. Astragalosides are the most widely studied secondary metabolites isolated from *Astragalus*, which support the integrity of the respiratory tract [5]. Astragaloside IV is the most widely known saponin due to its high biological potential as an antioxidant, antiaging, anti-inflammatory, antidiabetic, and antiatherosclerosis agent, in addition to being active against hepatitis virus B. Furthermore, polysaccharides isolated from many *Astragalus* species have a markedly positive effect on supporting the deep immune function by restoring the normal levels and functions of many immune cells, especially when the immune system is stressed by either environmental or endogenous challenges [6].

**Figure 1:**
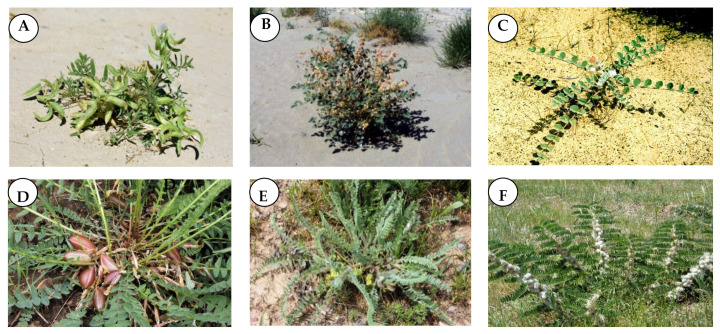
Photographs of some *Astragalus* species from Uzbekistan Flora (**A**): *A. campylotrichus* Bunge, (**B**): *A. chiwensis* Bunge, (**C**): *A. lehmannianus* Bunge, (**D**): *A. macronyx* Bunge, (**E**): *A. mucidus* Bunge, (**F**): *A. sieversianus* Pall (Photos: Alim Gaziev).

The aim of this study was to investigate the chemical composition of the volatile components obtained from the aerial parts of six species of *Astragalus*, namely, *A. sieversianus* Pall (*Asi*), *A. campylotrichus* Bunge (*Aca*), *A. lehmannianus* Bunge (*Ale*), *A. macronyx* Bunge (*Ama*), *A. mucidus* Bunge (*Amu*), and *A. chiwensis* Bunge (*Ach*) (Figure 1) grown in Uzbekistan, based on GC-MS analyses. Moreover, the metabolites’ differences between the species were explored by applying multivariate analyses based on the GC-MS analyses. In addition, the volatile extracts were assessed for their antioxidant and enzyme inhibitory activities.

## 2. Results and Discussion

### 2.1. Chemical Composition of Volatile Compounds

The volatile compounds obtained from the aerial parts of different *Astragalus* species were analyzed both qualitatively and semi-quantitatively by GC-MS. All volatile component mixtures were yellow in color with a characteristic odor. GC analyses of the essential oils revealed the presence of 105 metabolites (Table 1), which represented 73.28, 87.03, 74.38, 87.93, 85.83, and 91.39% of all *Aca*, *Ach*, *Ale*, *Ama*, *Amu*, and *Asi* volatile components, respectively.

The essential oils of different *Astragalus* species showed wide metabolic variation both qualitatively and quantitatively. Monoterpene hydrocarbons represented the major class solely in *A. sieversianus* (*Asi*), accounting for 68.47% of the total oil composition. On the contrary, monoterpene hydrocarbons were completely absent in *A. campylotrichus* (*Aca*). No oxygenated sesquiterpenes were detected in any species.

Sylvestrene was the major compound identified in *Asi, Amu* and *Ama*, with the highest concentration in *Asi* (64.64%), followed by *Amu* (14.14%) and *Ama* (10.63%). In *Ale*, (*E*)-2-hexenal (9.97%), sylvestrene (5.56%) and benzaldehyde (5.54%) represented the major identified components. n-Hexadecanoic acid (8.58%), phytol (7.49%) and 1-octen-3-ol (6.98%) were the main compounds detected in *Aca*. In *Ach*, (E)-2-hexenal (10.1%), butyl hexadecanoate ester (7.21%), *trans*-linalool oxide (6.81%) and butyl hexanoate (6.76%) were the predominant compounds. n-Hexadecanoic acid, trans-β–ionone, nonanal and benzaldehyde were detected in all *Astragalus* species, with the highest percentage in *Ach* for n-hexadecanoic acid and *trans*-β–ionone accounting for 8.58% and 3.34% of the whole essential oils, respectively. *Ama* and *Ale* contained the maximum amounts of nonanal (4.71%) and benzaldehyde (5.54%), respectively.

Many researchers have reported the essential oil compositions of various *Astragalus* species [7,8,9,10,11,12,13,14,15,16,17,18,19]. In almost all species, including our species of interest, the monoterpenes α-terpineol, its isomer 4-terpineol, γ-terpineol along with camphor, 1,8-cineole, α-pinene, and bornyl acetate are the most abundant monoterpenes. Additionally, other fatty acid components, such as hexadecanoic acid and its esters, are quite common in all the characterized species. These key components might be used as markers to confirm the identity of *Astragalus* species.

Our results are in accordance with previous work on *A. microcephalis*, which showed the presence of hexadecanoic acid, heneicosane, α-cadinene, and tridecanol as the major components [7]. In addition, the essential oils from dried aerial parts of *A. sericans* and *A. oocephalus* subsp. *stachyophorus* revealed α-pinene as the major component in *A. sericans*, whereas camphor, γ-terpineol, 1,8-cineole, and α-pinene were the major identified components of *A. oocephalus* subsp. *stachyophorus*, with their percentages being comparable to those in our oils.

### 2.2. Chemometric Analysis Based on GC-MS

**Figure 2:**
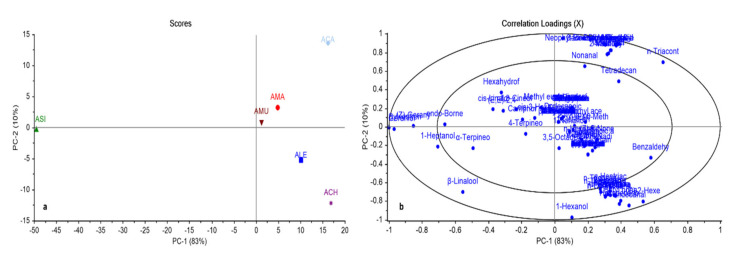
Principal component analysis score plot (**a**), correlation-loading plot (**b**) of GC-MS analysis of essential oils of different *Astragalus* species based on the identification of compounds shown in Table 1.

In general, chemometric analysis together with chromatographic techniques provide a successful tool for discrimination between closely related species [20,21]. In this study, both principal component analysis (PCA) and hierarchal cluster analysis (HCA) were applied to the relative peak areas of all identified compounds of different *Astragalus* species to find out the relationships between different *Astragalus* species and explore the similarities and differences between them. The PCA score plot and correlation-loading plot are shown in Figure 2a,b, respectively. In the PCA score plot, the impact percentage of the first two principal components covered 93% of the variance in the data. Different *Astragalus* species could be divided into four main groups. *Asi* was positioned rather remotely in the plot, highlighting an obvious dissimilarity from all other species. The closeness of *Ama* and *Amu* was evident, which fell in the same quadrant with *Aca*, but obviously segregated. *Ale* and *Ach* were grouped together in a quadrant, but rather distant from each other. In-depth inspection of the correlation-loading plot (Figure 2b) revealed the correlation between sylvestrene and geraniol, accounting for the complete separation of *Asi*, far away from the other *Astragalus* species. n-Triacontane and benzaldehyde were the key compounds accountable for the segregation of *Aca* and *Ale*. Regarding *Ach*, a strong relationship between (*E*)-2-hexenal, butyl hexadecanoate, and undecanal was observed, accounting for its separation from other *Astragalus* species. These results are presented in enlarged scale Figure S1 in the Supplementary Data. 

**Figure 3:**
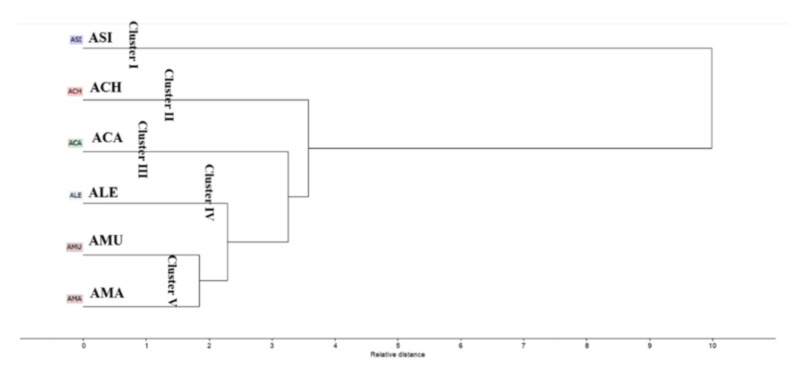
Hierarchical cluster analysis of the volatile components present in different *Astragalus* species based on the identification of compounds by GC-MS shown in Table 1.

The HCA dendrogram (Figure 3) categorized the *Astragalus* species into five main clusters. Cluster I, II and III displayed *Asi*, *Ach* and *Aca,* respectively. *Ale* was positioned in a separate cluster (IV), in close distance to *Aca*. Cluster V was subdivided into two sub-clusters of *Amu* and *Ama*, confirming the results from PCA.

**Figure 4:**
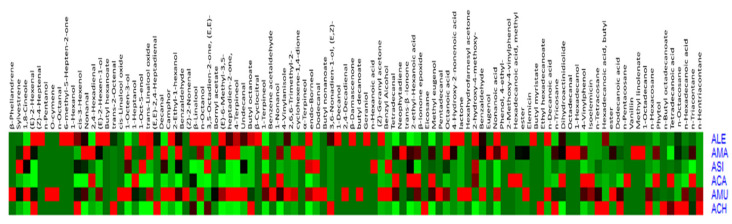
Heat map portraying the relative intensities of the various metabolites throughout different *Astragalus* species.

In addition, a heat map (Figure 4) was employed to demonstrate the distribution of all the data and to portray the relative intensities of the various metabolites throughout different species, which provides immediate visualization of information for easier understanding of the complexity of data. The colors indicate the relative content of each identified metabolite among the different species, as determined by the average peak response area by GC-MS.

This is the first report of a comparison of the chemical profiles of the volatile components present in the six species studied. No information regarding the classification of the *Astragalus* species in Uzbekistan has been available so far; therefore, this study could be used as the basis of future chemotaxonomical work.

### 2.3. Antioxidant Activity of Astragalus Species

The antioxidant potential of the essential oil samples was performed in vitro according to various assays, comprising phosphomolybdenum (PM), ferrous ion chelating (FIC), 2,2-diphenyl-1-picryl-hydrazyl-hydrate (DPPH), 2,2-azino bis (3-ethylbenzothiazoline-6-sulphonic acid) (ABTS) radical scavenging, cupric reducing antioxidant capacity (CUPRAC), and ferric reducing power (FRAP) assays.

The results displayed in Table 2 revealed that most of the samples showed considerable antioxidant potential in the assays. *A. mucidus* displayed the highest antioxidant activity (1.57 ± 0.08 mmol TE/g sample) in the PM assay followed by *A. lehmannianus* (1.31 ± 0.12 mmol TE/g sample), although without a statistically significant difference (*p* > 0.05). Regarding the DPPH assay, *A. macronyx* exhibited the maximum activity (24.12 ± 2.24 mg TE/g sample), followed by *A. lehmannianus* (21.90 ± 0.76 mg TE/g sample). *A. mucidus* (91.54 ± 1 mg TE/g sample) showed the highest antioxidant potential followed by *A. macronyx* (82.65 ± 4.94 mg TE/g sample) in the ABTS assay. Concerning the CUPRAC and FRAP assays, *A. lehmannianus* revealed the best activity, equivalent to 84.06 ± 0.57 mg TE/g oil and 49.47 ± 0.13 mg TE/g oil, respectively, followed by *A. macronyx*, which showed antioxidant potential of 80.28 ± 2.65 mg TE/g oil and 49.02 ± 2.32 mg TE/g oil. *A. campylotrichus* exhibited the most significant antioxidant power in the ferrous ion chelating assay (FIC, 51.69 ± 5.94 mg EDTAE/g sample), followed by *A. macronyx* (38.00 ± 0.88 mg EDTAE/g sample). Thus, it can be concluded that the essential oils from both *A. lehmannianus* and *A. macronyx* displayed the most significant antioxidant properties, as demonstrated by their prominent activities in most of the performed antioxidant assays. *A. chiwensis* exhibited moderate antioxidant activity in all reported assays.

Only a few studies have been found regarding the antioxidant properties of essential oils from *Astragalus* species, and most work has been carried out using the DPPH assay only. Ghahari et al (2018) determined the antioxidant properties of essential oil from *A. alopecurus* and found a significant DPPH radical scavenging ability (IC_50_ value: 146.59 µg/mL) [22]. In another study, the antioxidant abilities of two *Astragalus* (*A. oocephalus* subsp. *stachyophorus* and *A. sericans*) essential oils were investigated by lipid peroxidation, DPPH and reducing power assays. In the DPPH assay, the scavenging abilities were reported to be 134.22 and 44.43 µg/mL, respectively. In our study, we preferred to use the standard equivalent way for evaluating the antioxidant properties, rendering comparison with earlier studies difficult. However, the present results obviously represented the most comprehensive antioxidant profiling of the samples available so far.

### 2.4. Enzyme Inhibitory Activity of Astragalus Species

The enzyme inhibitory properties of the *Astragalus* essential oils were investigated against some enzymes, including acetylcholinesterase (AChE), butyrylcholinesterase (BChE), tyrosinase, amylase, and glucosidase. The results are shown in Table 3. The highest AChE inhibitory ability was provided by *A. sieversianus* with 4.55 mg GALAE/g oil, followed by *A. chiwensis* (4.52 mg GALAE/g oil), and *A. lehmannianus* (4.51 mg GALAE/g oil). No differences were observed for the AChE inhibitory abilities of three *Astragalus* species (*p >* 0.05). The lowest activity was obtained for *A. macronyx* in the AChE inhibition assay (4.01 mg GALAE/g oil). The BChE inhibition ability can be ordered as follows: *A. sierversinaus > A. mucidus > A. lehmannianus > A. chiewensis > A. macronyx.* Observed cholinesterase inhibitory activities could be attributed to the presence of some volatile compounds, including monoterpenes. This was also supported by several studies, which reported significant anticholinesterase abilities of several monoterpenes [23,24,25,26]. The strongest tyrosinase inhibition ability was determined for *A. lehmannianus* (138.42 mg KAE/g oil) and *A. macronyx* (132.14 mg KAE/g oil). In contrast to cholinesterase inhibition, the weakest tyrosinase inhibitory effect was recorded for *A. sieversianus* (118.20 mg KAE/g oil). The amylase inhibition abilities ranged from 0.76 mmol to 0.95 mmol ACAE/g oil in the following order: *A. chiwensis < A. sieversianus < A. campylotrichus < A. macronyx < A. mucidus < A. lehmannianus*. None of the tested essential oils were active against glucosidase.

To the best of our knowledge, no reports regarding the enzyme inhibition of *Astragalus* essential oils were available, only some studies on the enzyme inhibitory effects of extracts from the members of *Astragalus* [27,28,29]. The presented results could open new windows for potential medical applications of the members of *Astragalus.* Comparing our present results with those for *Ferula* essential oils, it was seen that the latter showed superior activity [30].

The biological effects of the *Astragalus* volatile components as enzymes inhibitors may be attributed to a high percentage of oxygenated compounds, such as alcohols, acids, aldehydes and ketones, which can bind non-selectively to amino and sulfhydryl groups of enzymes and cause a conformational change and, thus, loss of activity.

## 3. Materials and Methods

### 3.1. Plant Materials

Aerial parts of *Astragalus campylotrichus* Bunge (GN0555047), *A. macronyx* Bunge (GN0555205), *A. mucidus* Bunge (GN0555131), and *A. sieversianus* Pall. (GN0555089) were collected from the Tashkent region (Uzbekistan, in July 2015). *A. chiwensis* Bunge (N20160375) and *A. lehmannianus* Bunge (N20160328) were collected from the Karakalpak and Bukhara regions (Uzbekistan, in June 2016). The plants were identified by Dr. Orzimat Turginov at the Laboratory of Flora of Uzbekistan, and the voucher samples have been deposited at the Herbarium of the Botany Institute, Academy of Sciences of Uzbekistan.

### 3.2. Essential Oil Isolation

The plant samples were air-dried at a temperature not exceeding 30 °C and then powdered before use. Preparation of the volatile samples of the powdered *Astragalus* was performed by hydro-distillation for 2 h using a Clevenger-type apparatus. The oil samples were maintained at −30 °C in dark brown and air-tight, closed vials until their analyses. 

### 3.3. GC-MS Analysis

GC-MS characterization of volatile components was performed on an Agilent 7890 B gas chromatograph (Agilent Technologies, Rotterdam, The Netherlands) equipped with a VF-Wax CP 9205 fused silica column (100% polyethylene glycol, 30 m × 0.25 mm, 0.25 µm). It was coupled with a mass selective detector 5977A (Agilent Technologies). The instrumental settings and the separation conditions were as previously reported [31].

### 3.4. Chemometric Analysis

The data obtained from GC-MS were subjected to multivariate analysis. Principal component analysis (PCA) was performed as the first step in data analysis to provide an overview of all observations and samples and to identify and evaluate groupings, trends and strong outliers. Hierarchal cluster analysis (HCA) was used to allow clustering of samples. The clustering patterns were constructed by applying the complete linkage method. This presentation is more efficient when the distance between samples (points) is computed by the Euclidean method. Both PCA and HCA were achieved by utilizing Unscrambler® X 10.4 from CAMO (Computer Aided Modeling, Viken, AS, Norway) [20,32]. The heat map was constructed using Hierarchical Clustering Explorer 3.5 software (Human-computer interaction laboratory, University of Maryland, College Park, MD, USA).

### 3.5. Antioxidant and Enzyme Inhibitory Assays

Different protocols were performed to explain the antioxidant properties of the tested *Astragalus* oils. The protocols included reducing power (CUPRAC and FRAP), metal chelating, phosphomolybenum and free radical scavenging (DPPH and ABTS). Experimental details were as previously reported [33,34]. The same applies for inhibitory effects of the *Astragalus* oils tested against different enzymes (tyrosinase, amylase, glucosidase and cholinesterase) [33,35]. Both antioxidant and enzyme inhibition assays were explained by standard equivalents (trolox and EDTA for antioxidant; galantamine for cholinesterase; kojic acid for tyrosinase; acarbose for amylase and glucosidase activity). All samples were analyzed in triplicate in three independent experiments. The experimental details are given in the Supplementary Materials.

### 3.6. Statistical Analysis

Statistical analyses were performed using ANOVA experiment (with Tukey’s test, significant value: *p* < 0.05) and Xlstat 2017 software.

## 4. Conclusions

The volatile components identified from the aerial parts of six different *Astragalus* species revealed a significant variation between the species, as determined by GC-MS, which was confirmed by multivariate analysis. The essential oils showed relevant antioxidant as well as enzyme inhibitory activities. This study provides the first report about the biological activities of the studied species. *Astragalus* species can be utilized as promising natural sources for many medicinally important components, which could be used for drug development to address illnesses such as Alzheimer’s disease, diabetes mellitus and oxidative stress related diseases. Further studies are needed to test the in vivo activities.

## Figures and Tables

**Table 1:** Chemical composition of volatile compounds in the aerial parts of *Astragalus campylotrichus* (*Aca*), *A. chiwensis* (*Ach*), *A. lehmannianus* (*Ale*), *A. macronyx* (*Ama*), *A. mucidus* (*Amu*) and *A. sieversianus* (*Asi*).

	Rt	Compound	RI		Content (%)					
			Rep.	Cal.	*Aca*	*Ach*	*Ale*	*Ama*	*Amu*	*Asi*
1.	8.49	β-Phellandrene	1205	1203	-	-	-	-	0.25	-
2.	8.91	Sylvestrene	1205	1204	-	-	5.56	10.63	14.14	64.64
3.	9.42	1,8-Cineole	1208	1208	-	-	-	3.19	3.11	1.30
4.	9.58	Unidentified		1212	-	3.69	2.50	-	-	0.53
5.	9.81	(*E*)-2-Hexenal	1219	1219	-	10.1	9.97	4.57	4.42	-
6.	10.13	Unidentified		1230	-	-	1.32	-	-	0.50
7.	10.49	(*Z*)-4-Heptenal	1242	1242	-	-	1.22	-	-	-
8.	10.75	n-Pentanol	1250	1250	-	-	-	-	0.47	-
9.	11.3	o-Cymene	1268	1268	-	-	-	-	1.14	-
10.	11.89	n-Octanal	1287	1287	-	-	2.73	-	-	-
11.	13.37	6-Methyl-5-hepten-2-one	1335	1335	-	-	1.18	-	-	-
12.	13.76	1-Hexanol	1350	1350	-	5.27	3.09	1.12	2.07	2.13
13.	14.59	*cis*-3-Hexenol	1379	1379	-	-	0.63	1.58	0.72	0.48
14.	14.89	Nonanal	1389	1389	3.29	0.90	0.54	4.71	1.04	1.22
15.	15.04	2,4-Hexadienal	1395	1395	-	-	1.95	0.55	0.92	-
16.	15.22	(*E*)-2-Hexen-1-ol	1402	1401	-	-	0.92	-	-	-
17.	15.46	Butyl hexanoate	1410	1410	-	6.76	0.73	-	-	-
18.	15.8	*trans*-2-Octenal	1423	1423	-	-	-	-	0.42	-
19.	16.03	Unidentified		1430	-	-	0.73	-	-	-
20.	16.13	*cis*-Linalool oxide	1435	1435	-	-	-	0.55	0.50	0.31
21.	16.44	1-Octen-3-ol	1445	1446	6.89	-	-	0.39	1.11	-
22.	16.56	1-Heptanol	1452	1451	-	-	1.65	0.48	0.56	1.82
23.	16.74	1-Octen-5-ol	1458	1458	-	-	1.48	1.75	1.27	0.28
24.	16.91	*trans*-Linalool oxide	1466	1464	-	6.81	0.98	-	-	-
25.	17.3	Unidentified		1478	-	4.64	4.17	-	0.5	-
26.	17.49	(*E,E*)-2,4-Heptadienal	1486	1486	-	-	-	0.83	1.07	0.48
27.	17.7	Decanal	1494	1493	-	-	0.66	-	0.65	-
28.	17.96	Camphor	1505	1503	-	-	0.5	1.16	0.55	0.44
29.	18.04	2-Ethyl-1-hexanol	1509	1509	-	-	-	-	0.41	-
30.	18.2	Benzaldehyde	1513	1513	2.68	3.11	5.54	2.25	1.97	1.14
31.	18.58	(*Z*)-2-Nonenal	1530	1530	-	-	0.57	0.35	0.40	-
32.	18.94	β-Linalool	1542	1542	-	2.50	3.52	1.10	1.17	3.83
33.	19.17	n-Octanol	1552	1552	-	-	-	2.68	0.52	-
34.	19.43	(*E,E*)-3,5-Octadien-2-one	1562	1562	-	-	1.12	0.34	0.63	0.25
35.	19.87	Bornyl acetate	1579	1579	-	-	0.48	0.84	0.93	-
36.	19.98	(*E*)-6-Methyl-3,5-heptadien-2-one,	1582	1584	-	-	0.61	0.75	0.74	-
37.	20.22	4-Terpineol	1597	1594	-	-	0.35	-	0.91	0.25
38.	20.33	Undecanal	1598	1598	-	2.20	1.35	-	0.45	-
39.	20.56	Butyl octanoate	1610	1608	-	-	0.45	-	-	-
40.	20.61	β-Cyclocitral	1611	1610	1.98	-	-	1.43	1.02	-
41.	20.83	1-Terpineol	1621	1619	-	-	-	-	0.44	-
42.	21.06	Unidentified		1629	-	1.52	0.91	-	-	-
43.	21.16	Benzene acetaldehyde	1633	1634	5.78	-	1.40	3.45	2.8	1.11
44.	21.65	1-Nonanol	1655	1654	-	-	0.31	-	0.96	-
45.	21.98	4-Vinylanisole	1670	1668	-	-	-	1.02	0.6	-
46.	22.3	2,6,6-Trimethyl-2-cyclohexene-1,4-dione	1677	1682	-	-	-	0.48	-	-
47.	22.46	α-Terpineol	1688	1688	-	-	0.92	0.20	0.64	0.72
48.	22.52	endo-Borneol	1691	1691	-	-	-	-	0.82	0.67
49.	22.81	Dodecanal	1704	1703	-	-	-	-	0.36	-
50.	22.96	Butyl nonanoate	1714	1710	-	2.71	0.84	-	-	-
51.	23.66	3,6-Nonadien-1-ol, (*E,Z*)-	1731	1731	-	-	1.23	-	-	-
52.	23.78	Unidentified		1743	-	-	1.16	0.42	0.44	-
53.	23.97	1-Decanol	1756	1755	-	-	-	-	0.41	-
54.	24.98	2,4-Decadienal	1806	1800	-	-	0.49	-		-
55.	25.17	β-Damascenone	1815	1809	-	-		-	0.51	-
56.	25.21	Butyl decanoate	1821	1821	-	-	0.71	-	-	-
57.	25.75	Geraniol	1836	1836	-	-	-	-	-	0.45
58.	25.82	n-Hexanoic acid	1840	1840	-	-	1.28	0.16	1.75	
59.	25.96	(*Z*) -Geranyl acetone	1838	1846	-	-	-	-	0.34	0.46
60.	26.34	Benzyl Alcohol	1864	1864	2.80	-	-	1.13	1.21	0.49
61.	27.42	Tetradecanal	1911	1915	-	-	-	0.82	-	-
62.	27.49	Neophytadiene	1922	1922	2.38	-	-	0.98	0.62	0.78
63.	27.64	*trans*-β-Ionone	1926	1927	3.34	0.42	0.42	1.73	1.76	0.38
64.	27.96	2-Ethyl-hexanoic acid	1950	1943	-	0.63	-	-	-	-
65.	28.73	β-Ionone epoxide	1977	1980	2.32	-	-	0.96	0.98	0.5
66.	29.03	Eicosane	2000	1992	-	-	-	-	1.99	-
67.	29.17	Methyl eugenol	2002	2002	-	-	-	1.98	0.89	0.38
68.	29.54	Pentadecanal	2024	2021	-	-	3.40	0.87	-	-
69.	30.14	Octanoic acid	2052	2052	-	-	0.46	-	0.5	-
70.	30.34	4-Hydroxy-2-nonenoic acid lactone	2068	2062	-	-	-	-	0.36	-
71.	31.4	Hexahydrofarnesyl acetone	2114	2118	1.54	-	2.23	0.96	0.84	1.72
72.	31.62	2-Hydroxy-4-methoxy-benzaldehyde	2135	2130	-	-	0.56	0.52	1.79	-
73.	31.79	Unidentified		2139	1.57	-	-	0.70	0.96	0.63
74.	32.06	Eugenol	2151	2151	-	-	-	1.87	-	-
75.	32.15	Nonanoic acid	2158	2158	-	-	0.92	0.45	0.63	-
76.	32.25	4-ethyl- Phenol	2164	2164	-	-	-	1.03	-	-
77.	32.59	2-Methoxy-4-vinylphenol	2181	2182	2.92	-	-	-	-	-
78.	32.65	Unidentified		2154	4.56	-	-	0.58	0.71	-
79.	33.11	Methyl hexadecanoate	2208	2208	2.57	-	-	0.46	-	-
80.	33.21	Elemicin	2215	2215	-	-	-	0.36	1.84	-
81.	33.31	Butyl myristate	2229	2221	-	-	0.41	-	-	-
82.	33.78	Ethyl hexadecanoate	2246	2247	-	1.72	-	-	-	-
83.	34.07	n-Decanoic acid	2264	2264	-	-	0.58	-	0.55	-
84.	34.41	Unidentified		2283	3.44	-	-	0.42	0.46	-
85.	34.68	n-Tricosane	2300	2298	-	2.72	-	0.98	0.54	0.33
86.	35.12	Dihydroactinidiolide	2324	2322	1.6	-	0.73	0.66	0.52	0.34
87.	35.4	Octadecanal	2343	2340	-	-	-	0.32	-	-
88.	38.9	1-Hexadecanol	2365	2369	-	-	-	3.48	-	-
89.	36.08	4-Vinylphenol	2379	2379	3	-	-	1.37	1.25	-
90.	36.21	Isoelemicin	2390	2387	2.59	-	-	-	1.16	-
91.	36.37	n-Tetracosane		2396	-	3.81	-	-	1.08	0.28
92.	36.86	Butyl hexadecanoate	2419	2428	-	7.21	3.29	0.59	-	0.37
93.	37.67	Dodecanoic acid	2451	2453	-	-	-	0.43	1.57	-
94.	38.02	n-Pentacosane		2469	-	4.86	0.52	0.82	1.77	0.51
95.	38.81	Vanillin	2545	2544	1.61	-	-	-	-	-
96.	38.91	Methyl linolenate	2550	2551	-	-	-	0.97	-	-
97.	39.40	1-Octadecanol	2581	2585	-	-	-	7.89	-	-
98.	39.65	n-Hexacosane		2597	-	4.71	0.46	0.41	1.97	0.34
99.	39.74	Phytol	2603	2603	7.49	-	-	3.15	0.88	-
100.	40.20	n-Butyl octadecanoate		2632	-	2.13	-	-	-	-
101.	41.19	Tetradecanoic acid	2698	2697	9.92	4.53	1.47	0.15	1.95	1.72
102.	42.68	n-Octacosane		2794	-	5.32	0.87	1.09	-	0.56
103.	44.13	n-Hexadecanoic acid	2899	2898	-	3.61	0.47	0.65	1.67	0.29
104.	45.53	n-Triacontane	3000	2999	8.58	3.14	2.63	4.29	2.86	0.42
105.	46.80	n-Hentriacontane	3100	3099	-	1.86	-	-	0.92	-
Total					82.85	96.88	85.17	90.05	88.9	93.05
Monoterpene hydrocarbons					-	2.50	9.08	11.73	15.56	68.47
Oxygenated monoterpene					11.83	7.23	4.38	14.93	18.06	6.20
Sesquiterpene hydrocarbons					1.54	-	2.23	0.96	0.84	1.72
Oxygenated sesquiterpene					-	-	-	-	-	-
Alcohols					12.49	5.27	12.04	22.90	8.62	2.38
Aldehydes and ketones					15.61	16.31	30.56	20.81	11.38	5.20
Fatty acids and their esters					13.36	29.30	11.61	3.86	17.60	4.20
Others					69.48	87.15	69.48	62.43	53.90	16.66

Compounds were identified based on the compounds’ mass spectrometric data and retention indices in comparison with those of the National Institute of Standards and Technology (NIST) Mass Spectral Library (December 2011), the Wiley Registry of Mass Spectral Data, 8th edition. The content (%) was calculated using the normalization method.

**Table 2:** Antioxidant activities of the volatile extracts of *Astragalus* species according to the phosphomolybdenum (PM), 2,2-diphenyl-1-picryl-hydrazyl-hydrate (DPPH), 2,2-azino bis (3-ethylbenzothiazoline-6-sulphonic acid) (ABTS), cupric reducing antioxidant capacity (CUPRAC), ferric reducing power (FRAP), and ferrous ion chelating (FIC) assays.

Samples	PM (mmol TE/g Oil)	DPPH (mg TE/g Oil)	ABTS (mg TE/g Oil)	CUPRAC (mg TE/g Oil)	FRAP (mg TE/g Oil)	FIC (mg EDTAE/g Oil)
*A. sieversianus*	0.85 ± 0.09 ^b^	9.11 ± 0.33 ^d^	51.66 ± 2.42 ^e^	56.80 ± 0.25 ^c^	30.65 ± 2.08 ^c^	36.01 ± 0.46 ^b^
*A*. *mucidus*	1.57 ± 0.08 ^a^	15.95 ± 0.25 ^c^	91.54 ± 1.97 ^a^	72.46 ± 2.29 ^b^	38.11 ± 1.19 ^b^	18.11 ± 0.22 ^c^
*A*. *macronyx*	0.95 ± 0.04 ^b^	24.12 ± 2.24 ^a^	82.65 ± 4.94 ^b^	80.28 ± 2.65 ^a^	49.02 ± 2.32 ^a^	38.00 ± 0.88 ^b^
*A. lehmannianus*	1.31 ± 0.12 ^a^	21.90 ± 0.76 ^ab^	69.58 ± 5.04 ^c^	84.06 ± 0.57 ^a^	49.47 ± 0.13 ^a^	4.03 ± 0.41 ^d^
*A. chiwensis*	0.97 ± 0.17 ^b^	18.62 ± 1.36 ^bc^	57.84 ± 0.15 ^de^	70.73 ± 2.22 ^b^	39.03 ± 1.63 ^b^	11.74 ± 0.97 ^c^
*A*. *campylotrichus*	0.81 ± 0.07 ^b^	15.19 ± 1.53 ^c^	64.61 ± 1.74 ^cd^	67.78 ± 0.83 ^b^	39.27 ± 0.75 ^b^	51.69 ± 5.94 ^a^

Values expressed as means ± S.D. of three parallel measurements. TE: Trolox equivalent; EDTAE: EDTA equivalent. Superscripts within a column indicate significant differences in *Astragalus* species (*p* < 0.05).

**Table 3:** Enzyme inhibitory effects of the volatile extracts of *Astragalus* species.

Samples	AChE Inhibition (mg GALAE/g Oil)	BChE Inhibition (mg GALAE/g Oil)	Tyrosinase Inhibition (mg KAE/g Oil)	Amylase Inhibition (mmol ACAE/g Oil)
*A. sieversianus*	4.55 ± 0.09 ^a^	3.61 ± 0.33 ^a^	118.20 ± 3.53 ^d^	0.84 ± 0.04 ^a,b^
*A*. *mucidus*	4.48 ± 0.04 ^a^	3.61 ± 0.33 ^a^	124.75 ± 1.23 ^c^	0.90 ± 0.02 ^a^
*A*. *macronyx*	4.01 ± 0.13 ^b^	1.04 ± 0.09 ^d^	132.14 ± 0.81 ^b^	0.89 ± 0.04 ^a,b^
*A*. *lehmannianus*	4.51 ± 0.07 ^a^	3.12 ± 0.36 ^a,b^	138.42 ± 0.66 ^a^	0.95 ± 0.09 ^a^
*A*. *chiwensis*	4.52 ± 0.07^a^	2.79 ± 0.25 ^b,c^	132.79 ± 1.04 ^b^	0.76 ± 0.04 ^b^
*A. campylotrichus*	4.11 ± 0.15 ^b^	2.08 ± 0.34 ^c^	131.80 ± 1.11 ^b^	0.85 ± 0.09 ^a,b^

Values expressed as means ± S.D. of three parallel measurements. GALAE: Galanthamine equivalent; KAE: Kojic acid equivalent; ACAE: Acarbose equivalent. Superscripts within a column indicate significant difference in *Astragalus* species (*p* < 0.05).

## Supplementary Materials

The following are available online at https://www.mdpi.com/2223-7747/10/1/124/s1, Figure S1: Enlarged correlation-loading plot of GC-MS analysis of essential oils of different *Astragalus* species based on the identification of compounds shown in Table 1.
